# Supplementary material for: Uncertainty mapping and probabilistic tractography using Simulation-based Inference in diffusion MRI: A comparison with classical Bayes
Source: Med Image Anal. Author manuscript; Available in PMC 2026 Sep 8. (PMC7619459; doi:10.1016/j.media.2025.103580)
Supplement: Supplementary file 1 [file EMS218127-supplement-Supplementary_Material.pdf]

Supplementary Material

Mapping uncertainty for probabilistic tractography using Simulation-based Inference: A comparison with classical Bayes in diffusion MRI

Jose-Pedro Manzano-Patron[1], Michael Deistler[2], Cornelius Schröder[2],Theodore Kypraios[3]
Pedro Gonçalves[4], Jacob H. Macke[2,5], Stamatios N. Sotiropoulos[1]

[1]*Sir Peter Mansfield Imaging Centre (SPMIC), School of Medicine, University of Nottingham*
[2]*Machine Learning in Science, Excellence Cluster Machine Learning,*
University of of Tübingen & Tübingen AI Center
[3]*School of Mathematical Sciences, University of Nottingham*
[4]*VIB-Neuroelectronics Research Flanders (NERF)*
[5]*Department Empirical Inference, Max Planck Institute for Intelligent Systems, Tübingen*

MCMC implementation

We used MCMC implementation of FSL-bedpostx [7, 30, 31]. The likelihood is using the Ball&Sticks forward model and is defined assuming: a) independence between diffusion-sensitizing measurements $j = 1 : J$, b) additive zero-mean Gaussian noise with precision $\tau = 1/\sigma^2$. Gaussian noise is a good approximation for Rician MRI noise when the SNR is larger than 3 [88]. The likelihood function, $p(Y|\boldsymbol{\Omega}, \boldsymbol{\tau})$ is therefore:

$$p(Y|\boldsymbol{\Omega}, \tau) = \mathcal{N}(S, \sigma^2) \tag{5}$$

$$p(Y|\boldsymbol{\Omega}, \tau) = \prod_{j=1}^{J} \left(\frac{\sqrt{\tau}}{\sqrt{2\pi}}\right) \cdot \exp\left(-\frac{\tau}{2}\sum_{j=1}^{J}(Y_j - S_j)^2\right), \tag{6}$$

where Y_j is the measured signal with gradient j, Ω are model parameters of interest, and S_j is the predicted signal by the forward model, given by the Ball&Sticks model (see eq. 3 and eq. 4 in the main text). Noise variance is marginalised out using reference priors (so that it is not inferred), leading to a likelihood dependent only on model parameters Ω [21]:

$$p(Y|\boldsymbol{\Omega}) \propto \left(\sum_{j=1}^{J} \frac{(Y_j - S_j)^2}{2}\right)^{-\frac{J}{2}} \tag{7}$$

Regarding the prior distributions of the parameters, the prior on spherical angles ensures a uniform prior on the sphere, while positivity constraints are used on S_0, d, and f_n [21].

$$\begin{gathered} p(\theta_n, \phi_n) \sim \mathcal{U}(0, 2\pi)|_{cart} \rightarrow \frac{|\sin\theta_n|}{2}|_{sph} \\ p(f_1) \sim \mathcal{U}(0,\, 1) \\ p(d) \sim \mathcal{U}(0,\, 1) \\ p(S_0) \sim \mathcal{U}(0,\, \infty) \end{gathered}$$

In case of multiple fibre compartments, the sum of all anisotropic volume fractions is constrained to be in [0,1]. In addition, Automatic Relevance Determination (ARD) priors are used for the corresponding volume fractions f_n, $n \geq 2$. ARDs are shrinkage priors that a-priori heavily penalise model complexity and the existence of extra compartments, providing high support for near to zero values for volume fractions f_n, $n \geq 2$. They therefore allow indirect model selection to be performed, when the most complex model is fitted, with low prior support for multiple compartments. As indicated in [7], ARD priors are $p(f_n) \sim 1/f_n \quad (n \geq 2)$.

A univariate Random-walk adaptive Metropolis-Hastings algorithm is used for MCMC. Model parameters are initialised by a non-linear deterministic fit (using Levenberg-Marquadt non-linear optimisation). Proposals for MCMC are univariate Gaussians, centred in the last accepted sample and with adaptive

variance to ensure a balanced acceptance/rejection ratio of 50%. MCMC runs for 1,000 burn-in iterations that allow the chain to converge and subsequently 1,250 samples are obtained for each parameter. To avoid autocorrelation in the Markov chain, one of every 25 samples is retained (thinning=25). Hence, after thinning and burn-in, the approximated posterior distribution of each model parameter contains 50 samples.

Uniform sampling on the sphere

Linear and spherical geometries obey different principles. A uniform sampling in the plane does not return a uniform sampling in the sphere, as there is a different surface area for the same number of points. Close to the poles of the sphere, the differential surface area element gets smaller (it shrinks), and the wrapping of the uniform planar distribution produces an oversampled area. In contrast, close to the equator, the differential surface area increases (it stretches) and the density of points is lower (see Suppl. Fig. S1A). Thus, we should include fewer points near the poles and more points near the equator to achieve a uniform distribution on the sphere. We apply the following correction to achieve uniform sampling in the sphere when training and evaluating the SBI framework (see Suppl. Fig.S1B):

$$\theta \sim 2\pi \cdot \mathcal{U}(0,1) \tag{8}$$

$$\phi \sim \cos^{-1}(1 - 2 \cdot \mathcal{U}(0,1)) \tag{9}$$

Estimating mean fibre orientations and their uncertainty

In order to obtain the mean fibre orientation of M vector samples described by spherical angles (θ, ϕ), we use the average dyadic tensor [5]. After a spherical to cartesian coordinates conversion, the dyadic tensor corresponding to a vector $\hat{\mathbf{v}}_m$ is the outer product of the vector with itself. The average dyadic tensor $\mathbf{V}$ is the mean over the M samples:

$$\mathbf{V} = \frac{1}{M} \sum_{m=1}^{M} \mathbf{v}_m \mathbf{v}_m^\top \tag{10}$$

The principal eigenvector $\mathbf{e}_1$ of the mean dyadic tensor $\mathbf{V}$ (i.e. the one corresponding to the largest eigenvalue) represents the mean orientation of the set of M vector samples. The corresponding eigenvalue provides a measure of how concentrated the orientations are around that direction $\mathbf{e}_1$. Hence, the orientation uncertainty can be computed as:

$$uncertainty = 1 - \text{EigMax}(\mathbf{V}) \tag{11}$$

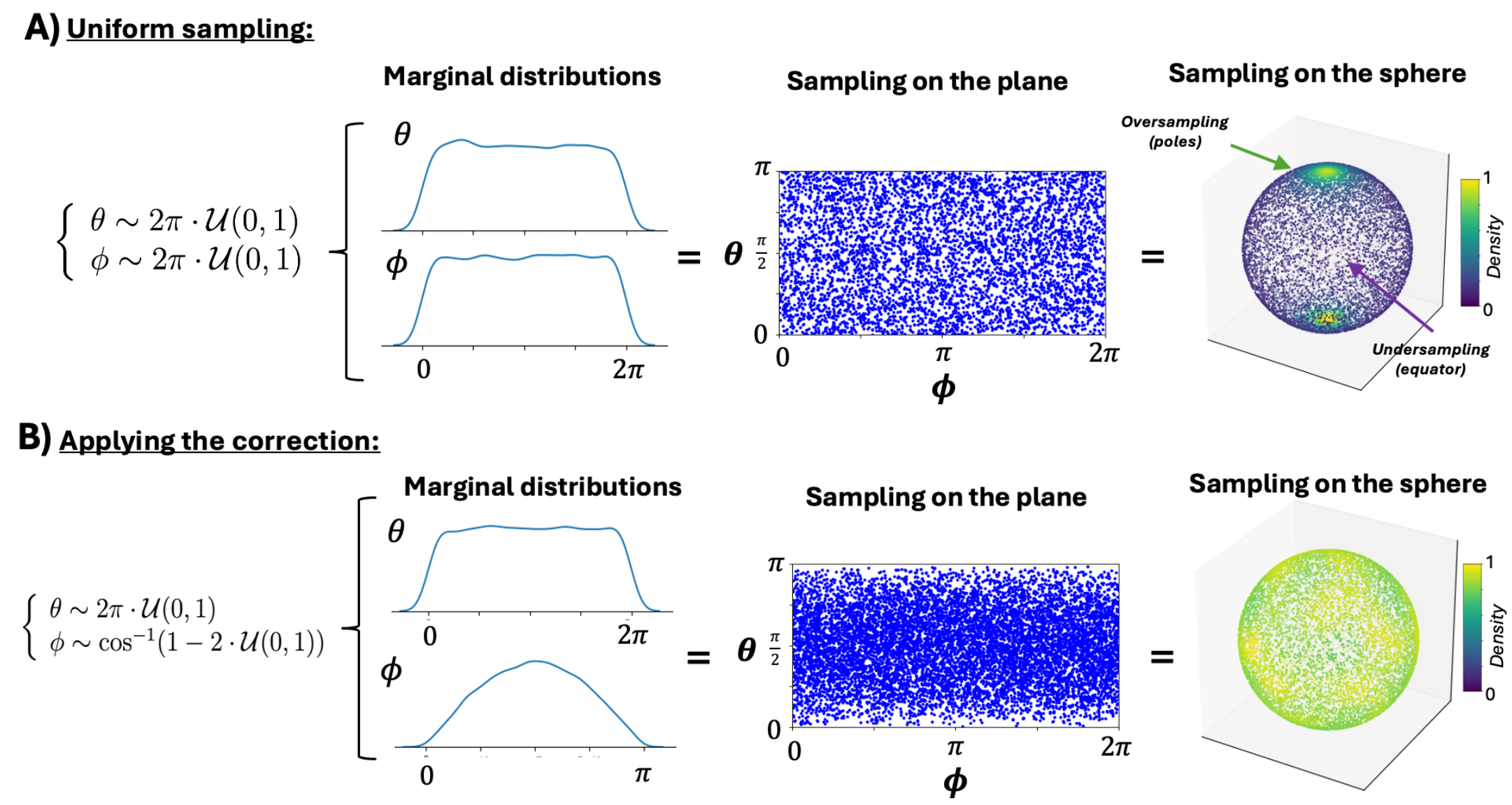

Figure S 1: **Defining uniform priors on the sphere for fibre orientations**. A) Uniform sampling of orientations in the interval (0, 2π) leads to uniform coverage on the plane, but not on the sphere, as it produces oversampling at the poles and undersampling at the equator. B) Applying the correction, uniform sampling on the sphere can be achieved, even if this does not look uniform on the plane $\theta - \phi$. In both A) and B), from left to right: (i) Definition of the prior distributions of the fibre orientations, (ii) Marginal distributions produced when sampling from such priors, (iii) joint distribution represented on the $\theta - \phi$ plane, (iv) joint distribution represented on the sphere, colour-coded by the density of the samples.

Since $\mathbf{v}_m$ are unit vectors, $\mathbf{V}$ is symmetric and positive semi-definite. The eigenvalues of $\mathbf{V}$ represent the spread or concentration of the orientation samples. If the orientations are perfectly aligned, $\text{EigMax}(\mathbf{V}) = 1$, and the other eigenvalues are zero. This corresponds to no uncertainty (uncertainty $= 0$). On the other hand, for completely isotropic distributions (uniformly sampled over the sphere), the eigenvalues of $\mathbf{V}$ are equal, and the largest eigenvalue is $1/3$. This gives the highest uncertainty ($uncertainty = 1 - \text{EigMax}(\mathbf{V}) = 1 - 1/3 \approx 0.66$).

Accuracy and Uncertainty Metrics

To provide quantitative performance metrics, a reference value is generally needed. For comparison of the mean estimates in synthetic data, these reference values are the ground-truth parameter values used to simulated the dMRI signals. However, there is no ground-truth for uncertainty, nor for estimates in in-vivo brain data. In those cases, we compared the SBI estimates against the estimates from the established MCMC implementation in FSL-bedpostX, i.e. the latter are used as the reference values.

Accuracy The agreement between scalar parameters is generally indicated as the linear Pearson's

correlation r between the reference values and the mean of the estimated posterior distribution samples, while errors are calculated as the % of their difference:

$$e_{\omega_p}[\%] = 100 \times \frac{\omega_p - \hat{\omega}_p}{\omega_p} \tag{12}$$

For fibre orientations, the angular difference $\angle e$ was defined as the crossing-angle between the reference value $\mathbf{v}_n$ and the mean estimated $\hat{\mathbf{v}}_n$ from the posterior samples:

$$\angle e_n[^\circ] = cos^{-1}\langle \mathbf{v}_n \cdot \hat{\mathbf{v}}_n \rangle), \tag{13}$$

where $\hat{\mathbf{v}}_n$ is the dyadic vector, i.e. the principle eigenvector of the dyadic tensor (see section above). In the case of multiple fibre orientations, we matched the closest pairs before calculating the errors per orientation.

For model selection, we calculated the confusion Matrix (CM) of the actual number of fibres N vs predicted number of fibres $\hat{N}$ for each approach. This offered not only the detection rates for each fibre but also potential trends and patterns in the mis-classified cases.

For tractography evaluations, for each estimated tract, the corresponding spatial distribution was thresholded (≥ 0.001) to remove noisy tails. To compare a pair of corresponding tract reconstructions, we calculated the Pearson's correlation between the respective spatial distributions (after vectorising each of them). To avoid bias from any of the tract reconstructions in comparison, the correlation was performed within a population-based atlas-derived tract mask, available from [71].

Uncertainty For scalar parameters, the standard deviation (width) of the posterior distribution was used to obtain a metric of uncertainty. For fibre orientations, the maximum eigenvalue λ_n of the dyadic tensor of the posterior orientation samples was used to provide a measure of uncertainty, i.e. how concentrated the orientations were around the mean direction of the samples. In synthetic data, we evaluated how such orientation uncertainty changed as a function of noise level and training data size, as uncertainty is expected to reduce as the SNR and/or the training data size increase (see also [55]). In real data, we used MCMC uncertainty maps as reference for direct comparisons, and the probabilistic tractography results as indirect indications of how consistent spatially-propagated uncertainty were.

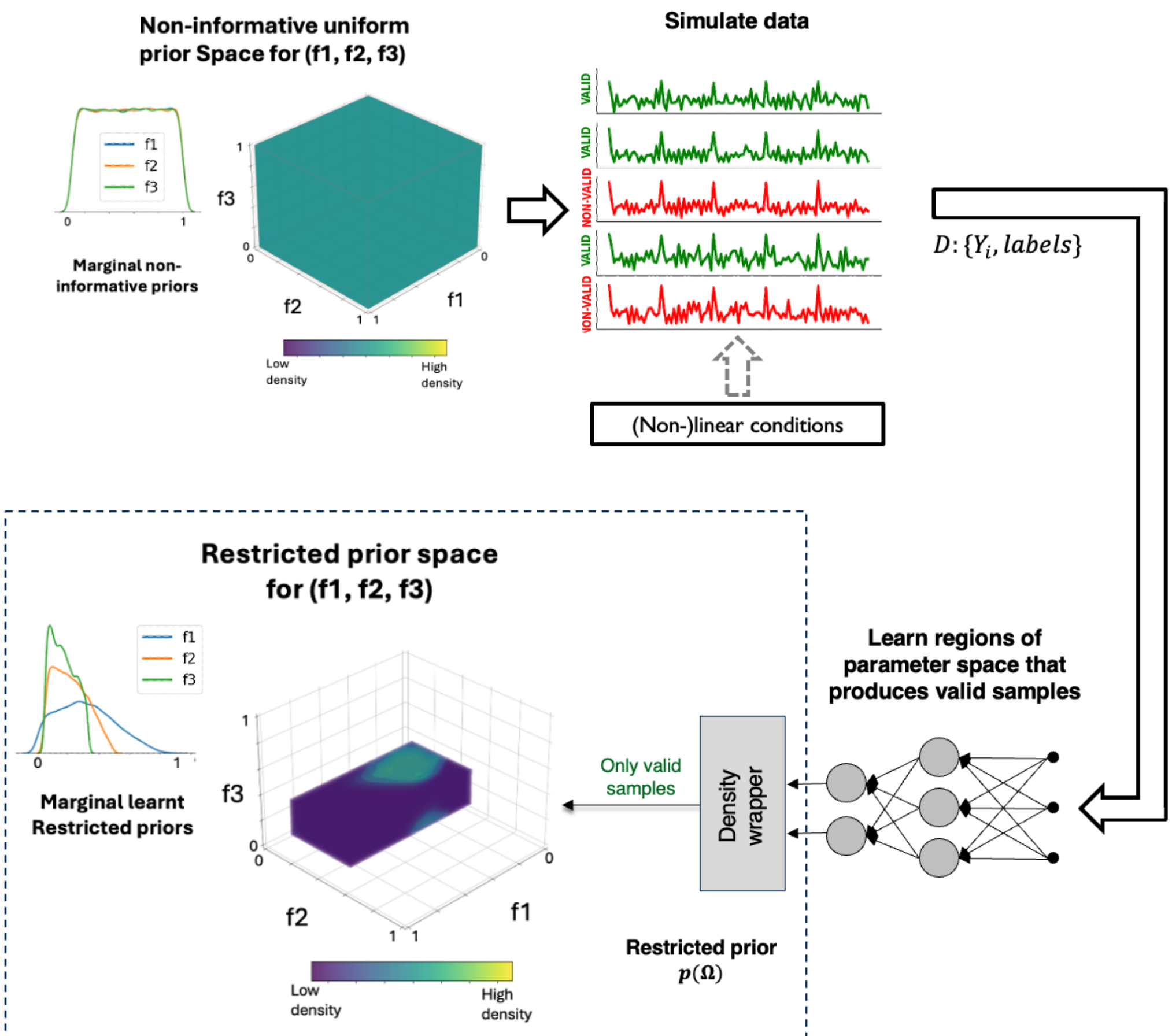

Figure S 2: **Restricted priors constraining the parameter space** compared to non-informative uniform prior sampling. We simulated data Y using samples from the non-informative uniform priors and tagged these as "valid" or "non-valid" (according to the constraints we wanted to enforce) to create our training dataset D. A classifier was then trained to learn what is the signal stemming from "valid" parameter combinations and was used to produce samples only from the regions of the parameter space of interest i.e. a restricted prior. The figure shows an example of the learnt restricted priors used in this paper, which reflect the conditions and non-linear boundaries specified in Table 1 and target the high density areas; in this case, the marginal (top 2-D histograms) and the 3-D joint space of (f_1, f_2, f_3) is shown. For a 3-fibre model, the sampling efficiency of prior samples is also increased with speed-ups of 105% compared to using a uniform sampling scheme and reject using the constraints as a post-sampling step.

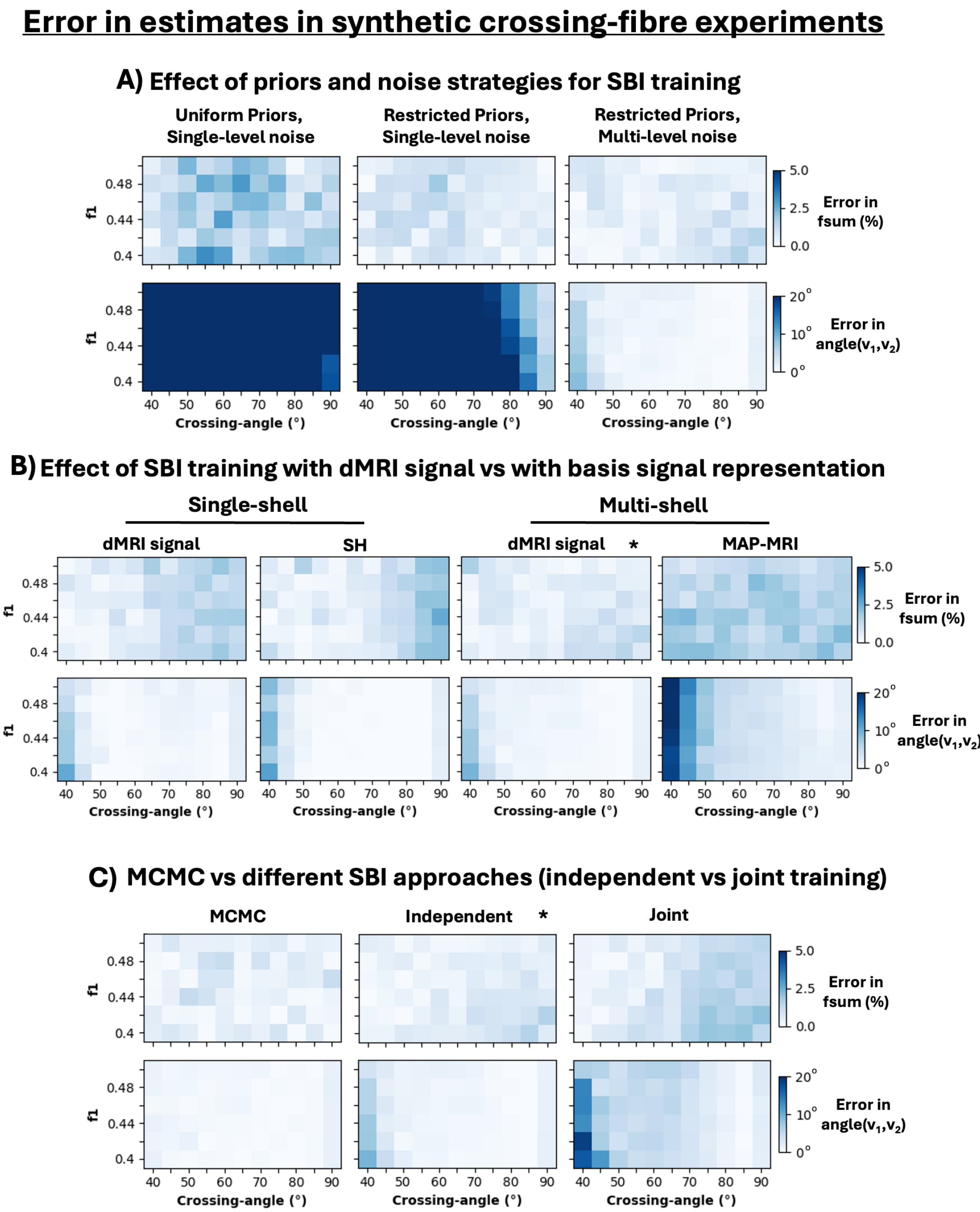

Figure S 3: **Evaluating accuracy of different SBI designs in synthetic fibre crossings ($N = 2$).** dMRI was synthetically generated for patterns with two crossing fibres, varying the anisotropic volume fraction f_1 and their crossing angle (rest of parameters were $d = 0.0012\ mm^2/s$, $f_2 = 0.2$, $SNR = 25$ for all experiments). Median error over 100 noisy realisations, where the error is measured as $100 * (groundtruth_i - estimate_i)/groundtruth_i$ [%], are shown for the same cases as in Fig. 3: A) Different prior definition and noise strategies for SBI training. B) SBI trained with raw dMRI signal (acquisition-specific) vs a basis set signal representation (acquisition-agnostic). C) Comparing MCMC fitting a model with $N = 2$ fibres against NPEs trained only with 2-fibres examples (Independent) or with a joint superset that contains both 1- and 2-fibres cases (Joint).

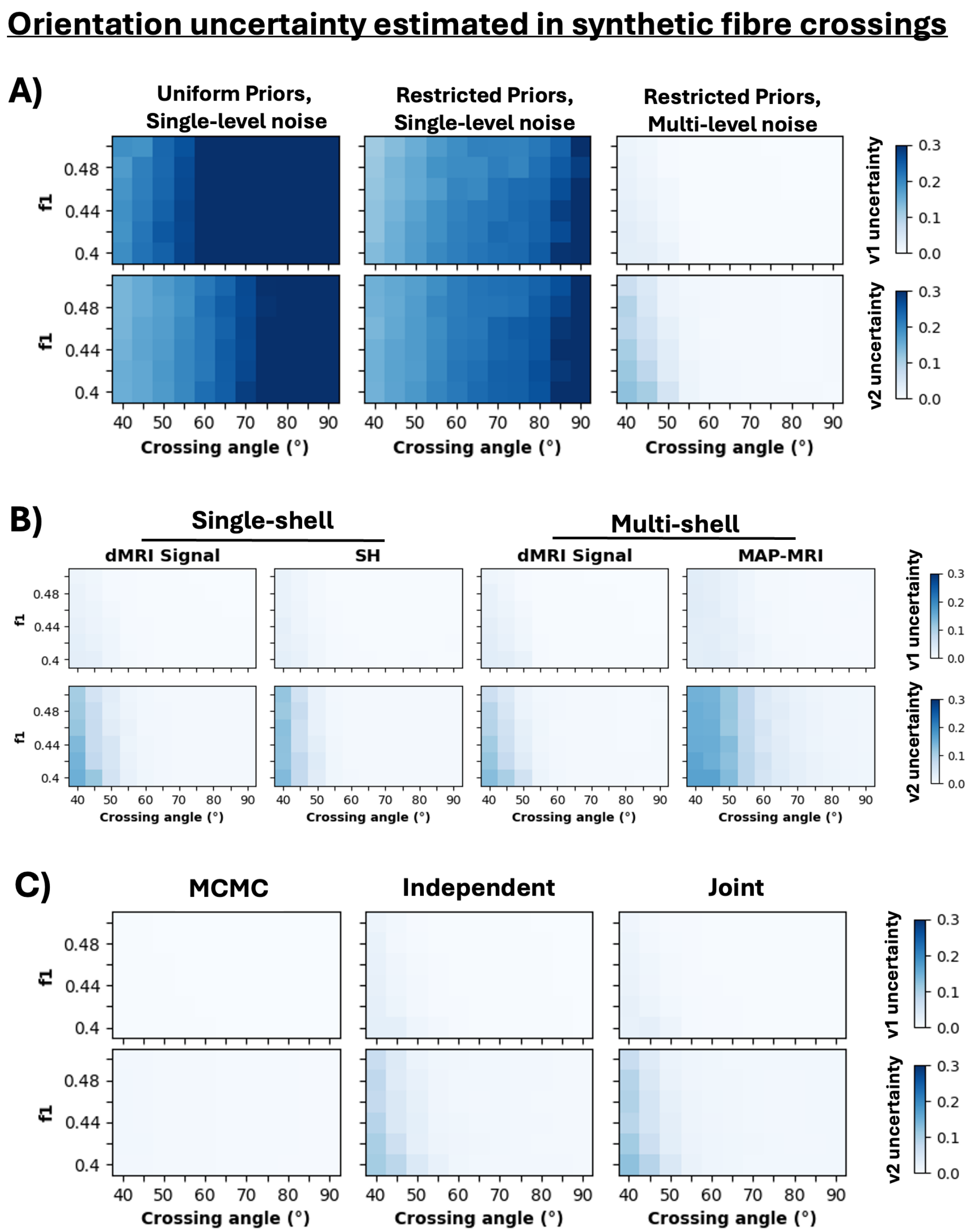

Figure S 4: **Evaluating precision of different SBI designs in mapping orientation uncertainty in synthetic fibre crossings ($N = 2$).** dMRI was synthetically generated for patterns with two crossing fibres, varying the anisotropic volume fraction f_1 and their crossing angle (rest of parameters were $d = 0.0012\ mm^2/s$, $f_2 = 0.2$, $SNR = 25$ for all experiments). Uncertainty in $\mathbf{v}_1$ and $\mathbf{v}_2$ obtained from the width of the respective posteriors (median over 100 noisy realisations) are shown for the same cases as in Fig. 3 in the main text: A) Different prior definition and noise strategies for SBI training. B) SBI trained with raw dMRI signal (acquisition-specific) vs a basis set signal representation (acquisition-agnostic). C) Comparing MCMC fitting a model with $N = 2$ fibres against NPEs trained only with 2-fibres examples (Independent) or with a joint superset that contains both 1- and 2-fibres cases (Joint).

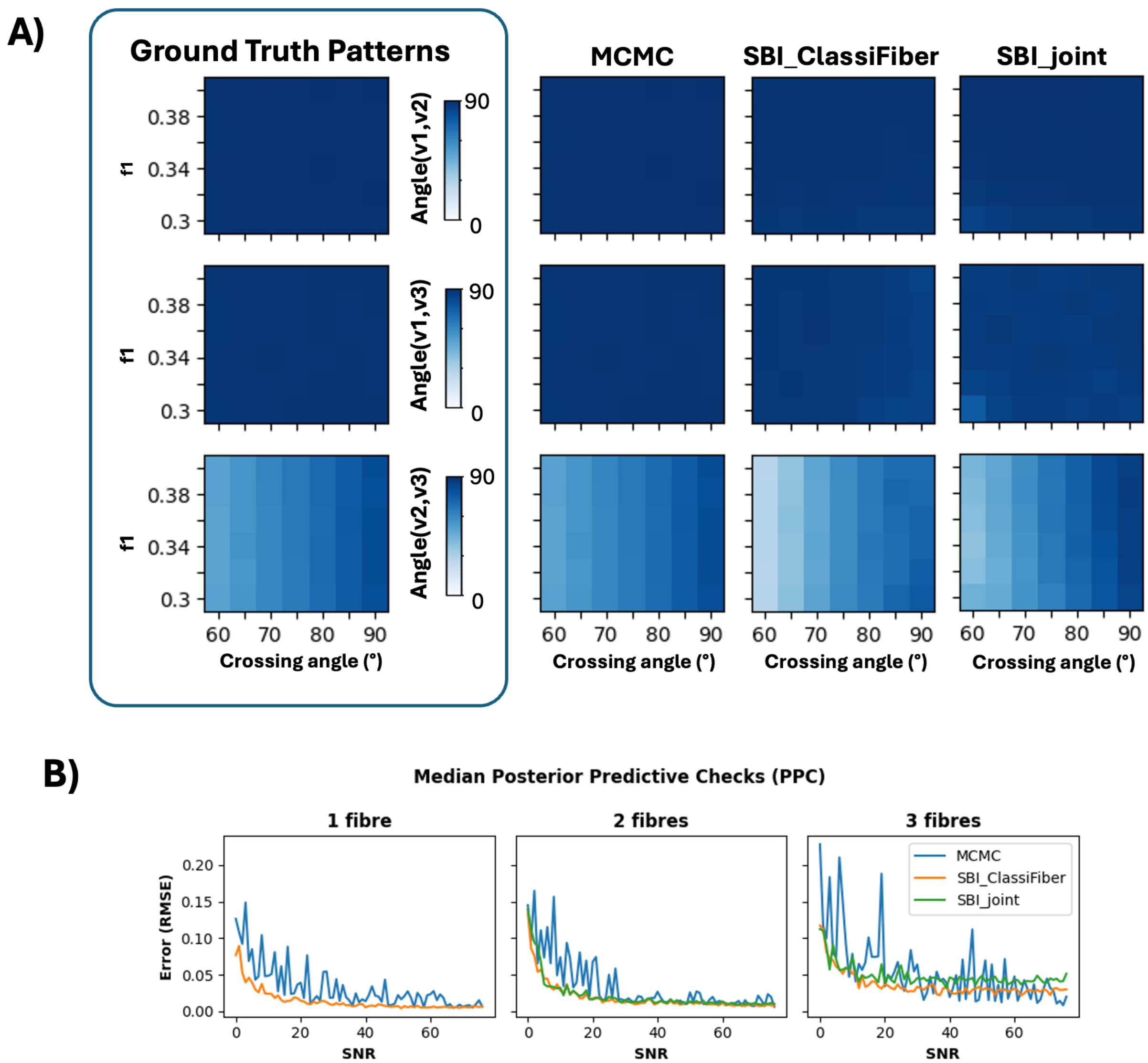

Figure S 5: **Evaluating performance of different SBI designs and MCMC in estimating crossing angles in synthetic 3-way fibre crossings** ($N = 3$)**.** A) Estimates from three approaches for synthetic data, where the first fibre orientation was perpendicular to the rest of compartments, and f_1 and the crossing angle between the second and third fiber were varied. The rest of the parameters were kept to $d = 0.0012$, $f_2 = 0.25$, $f_3 = 0.15$, $SNR = 25$. B) Posterior Predictive Checks (PPC) as a function of SNR for synthetic experiments with different fiber complexities ($N = 1, 2, 3$) and parameter combination. Each plot shows the relative error (median across 1,000 experiments) of the model predicted signal (using the mean posterior value for each parameter) with respect to the true signal.

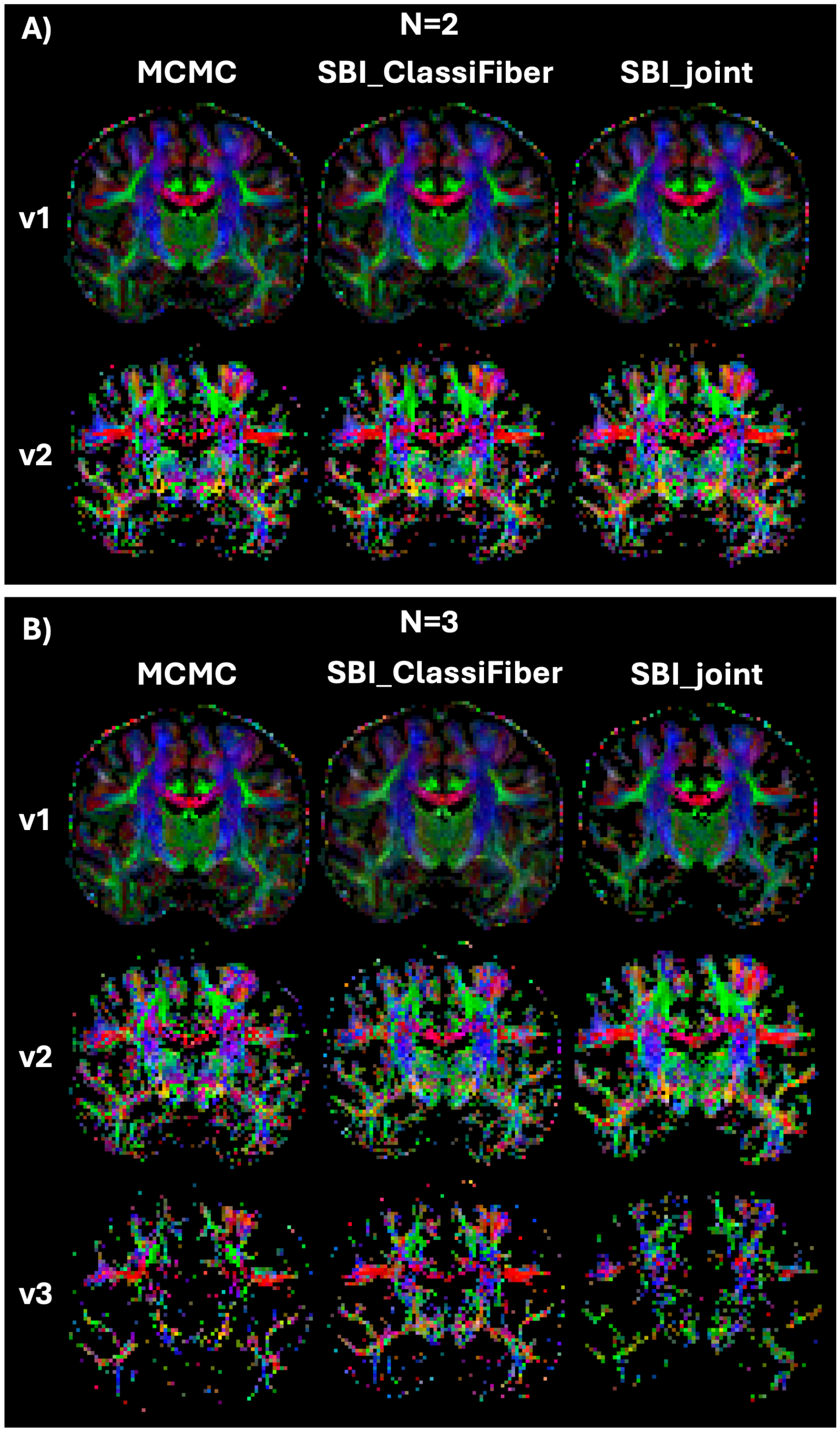

Figure S 6: **Maps of fiber orientation estimates (mean orientations of the posterior distributions) in in-vivo brain data obtained using MCMC, SBI_ClassiFiber and SBI_joint.** A) Multi-shell 2-fibres model. B) Multi-shell 3-fibres model. In SBI, all samples have been corrected to avoid compartment swapping, as detailed in Fig. 7 in the main text.

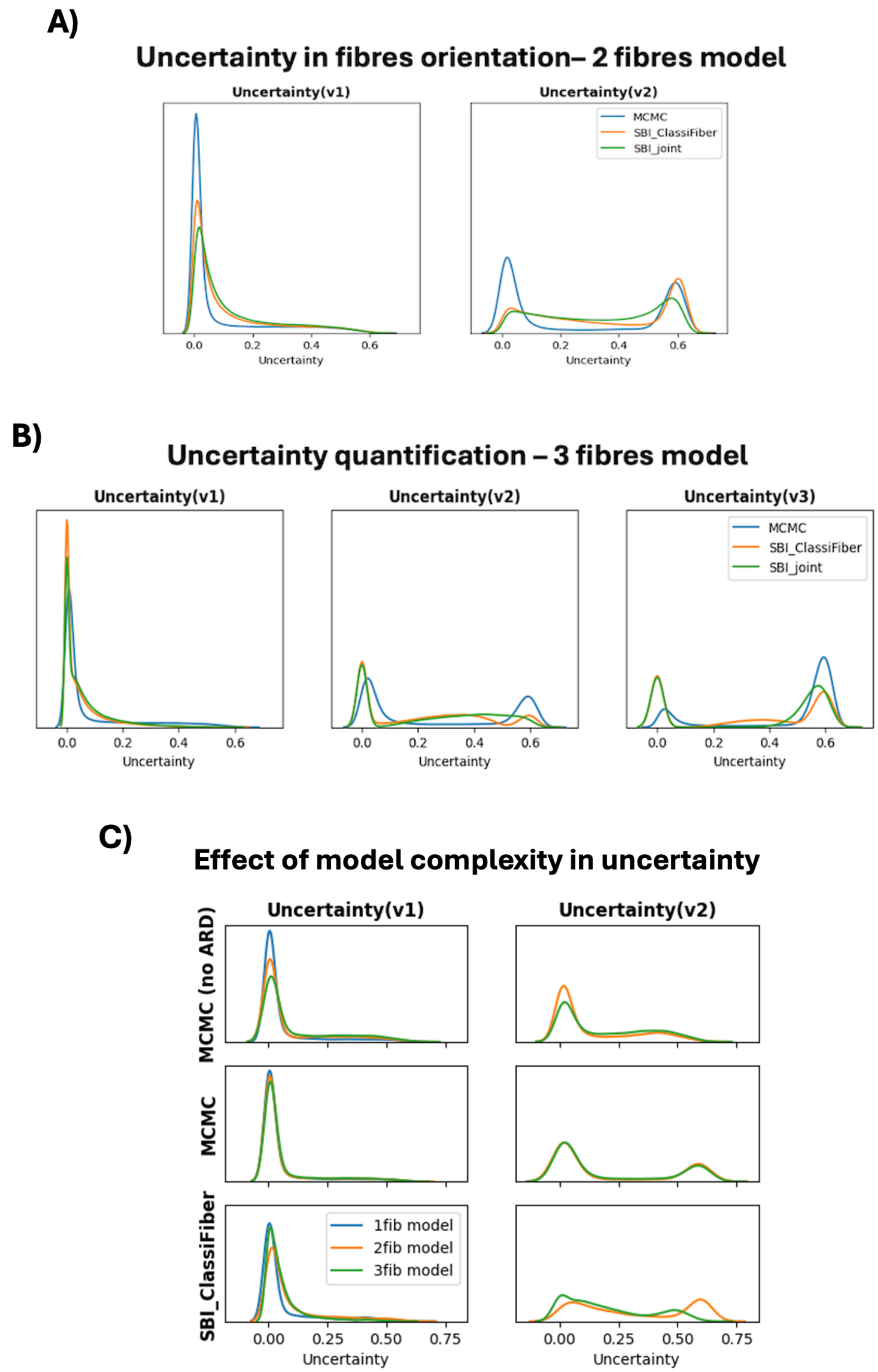

Figure S 7: **Comparison of uncertainty histograms returned by the different approaches** A) Uncertainty distribution in v_1 and v_2 mapped by the different approaches in the 2-fibres model. B) Uncertainty distribution in v_1, v_2 and v_3 mapped by the different approaches in the 3-fibres model. C) Effect of increasing model complexity (models with N 1 to 3) in the uncertainty quantification of the first (v_1) and second (v_2) fibre orientation. Note the effect of the ARD priors in MCMC (first and second rows).

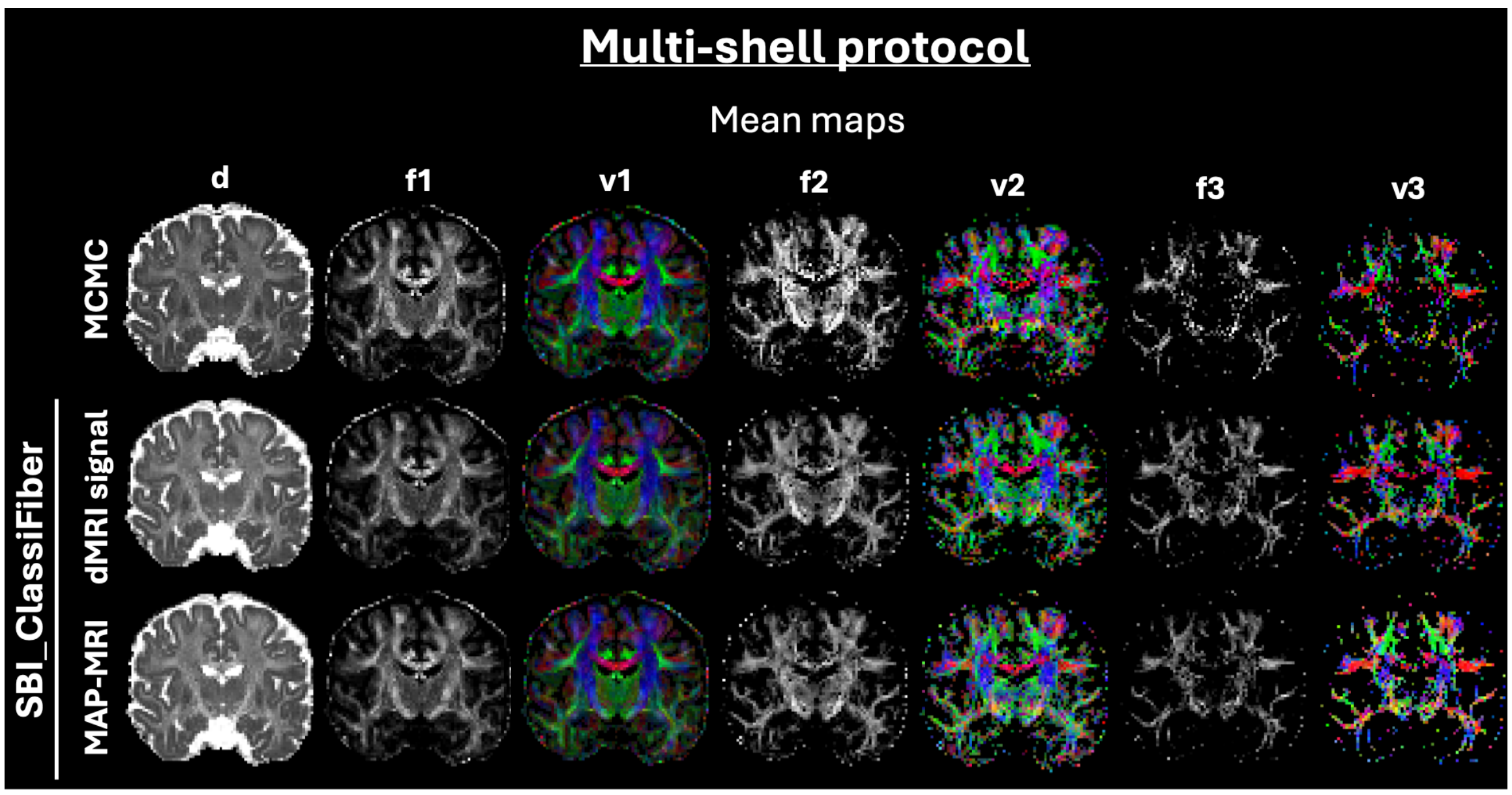

Figure S 8: **Maps of mean posterior estimates in in-vivo data from SBI trained against the raw dMRI signal (acquisition-specific) or a basis representation of the signal (acquisition=agnostic)**, for $N = 3$ compartments. MCMC estimates are shown for reference at the top row. Multi-shell data have been used and a MAP-MRI basis. NPEs are trained with 4 million samples and with the SBI_ClassiFiber architecture.

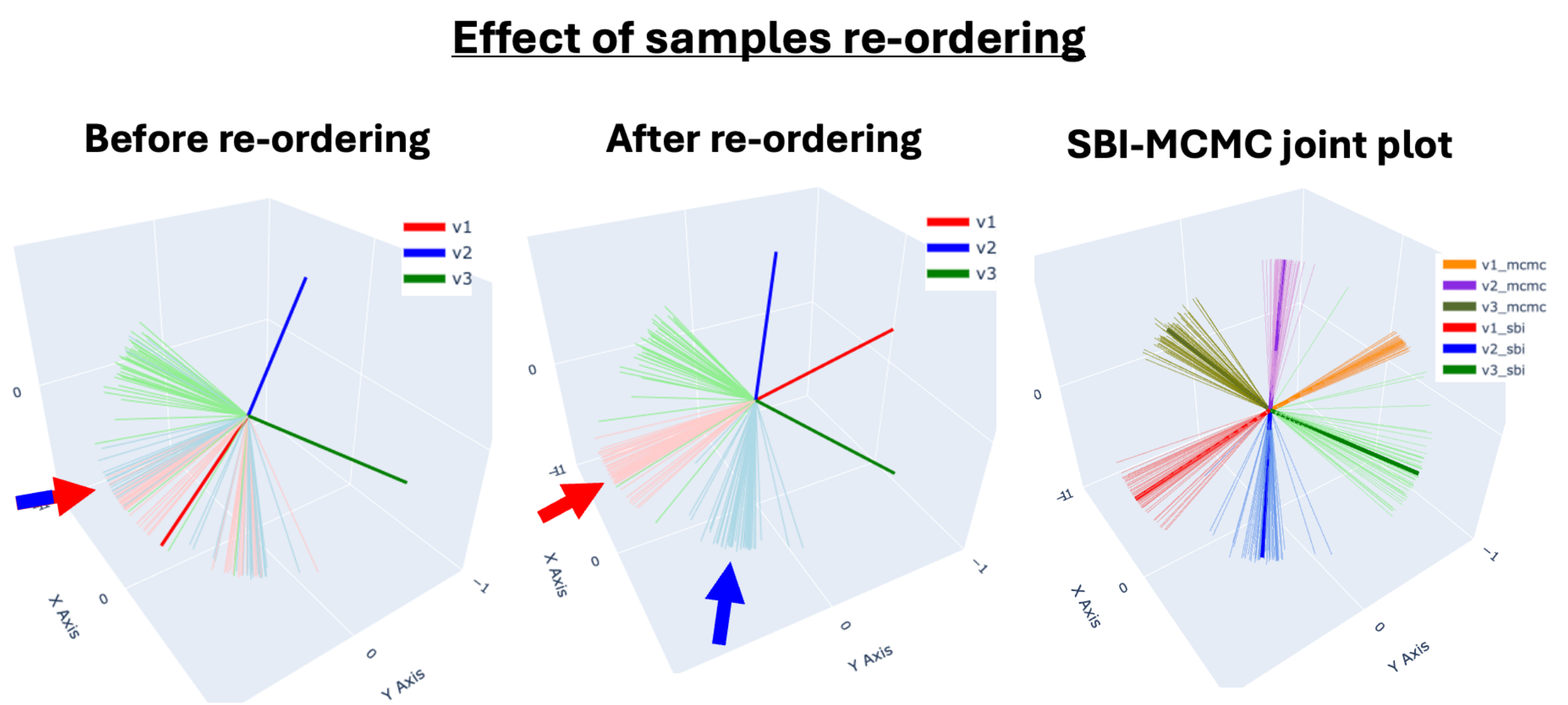

Figure S 9: **Effect of sample re-ordering in the joint distribution of fibre orientation samples**. Example of a 3-way crossing obtained from in-vivo brain data in the Centrum Semiovale. Left: samples from the posterior provided by SBI. Middle: SBI samples re-grouped, based on similarity of orientations. Right: joint plot of the fibre orientation samples provided by MCMC and SBI after re-ordering. In all cases, samples represent the same posterior distribution, but the re-ordering of SBI samples helps in matching the mean vectors obtained by MCMC.

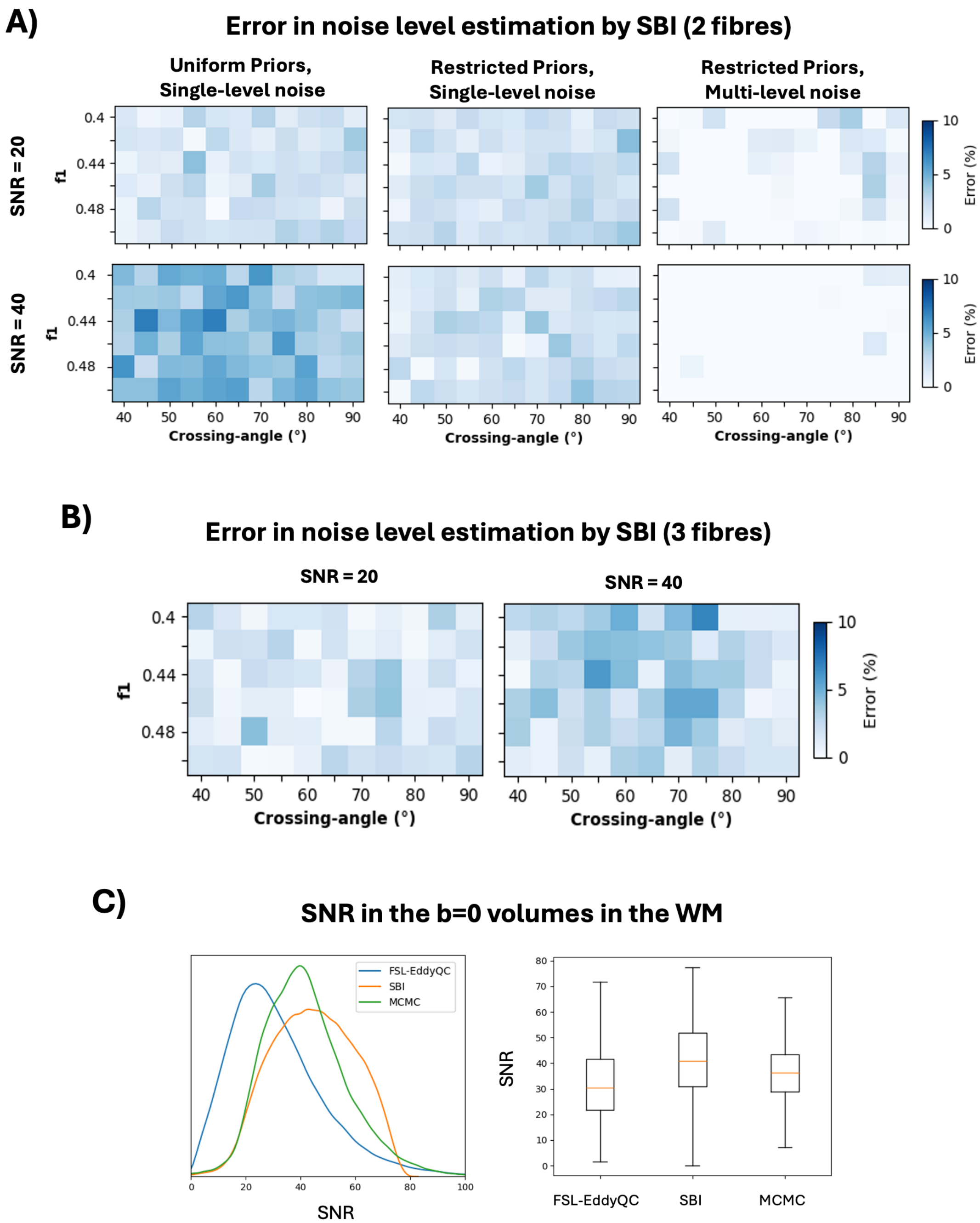

Figure S 10: **Noise estimation by SBI.** A) Comparison of the error (in %) of the mean estimated SNR level by the different SBI designs, using the same models (i.e. $N = 2$) and synthetic data as in Fig.3 of main text at different noise levels. B) Error (in %) of the mean estimated SNR level using the same dataset as in panel A) but doing inference using the SBI_Classifiber with $N = 3$ (i.e. including model selection). C) Comparison of the distribution of mean SNR estimates in white matter obtained by MCMC and SBI, compared against SNR estimated directly from the b=0 s/mm^2 volumes by FSL-EddyQC [82].
